# Hemobilia caused by a ruptured hepatic cyst: a case report

**DOI:** 10.1186/1752-1947-5-26

**Published:** 2011-01-20

**Authors:** Viplove Senadhi, Deepika Arora, Manish Arora, Sudhir Dutta

**Affiliations:** 1 Johns Hopkins University/Sinai Hospital Program in Internal Medicine, Sinai Hospital, Baltimore, MD, USA; 2 Elmhurst Hospital/Mount Sinai School of Medicine, New York, NY, USA; 3 Division of Gastroenterology, University of Maryland School of Medicine and National Institute of Health (NIH), Baltimore, MD, USA; 4 Division of Gastroenterology, Department of Internal Medicine, Johns Hopkins University/Sinai Hospital Program, Sinai Hospital, Baltimore, MD, USA; 5 University of Maryland School of Medicine, Baltimore, MD, USA

## Abstract

**Introduction:**

Hemobilia is a rare cause of upper gastrointestinal bleeding. More than 50% of hemobilia cases are related to iatrogenic trauma from hepatobiliary procedures, and needle biopsy of the liver represents the most common cause. A minority of hemobilia cases are due to hepatobiliary disorders such as cholangitis, hepatobiliary cancers, choledocholithiasis, and vascular abnormalities in the liver. The classic presentation of hemobilia is the triad of right upper quadrant (biliary) pain, obstructive jaundice, and upper gastrointestinal bleeding. We report a rare case of hemobilia caused by a spontaneous hepatic cyst rupture, where our patient presented without the classical symptoms, in the absence of therapeutic or pathological coagulopathy, and in the absence of spontaneous or iatrogenic trauma.

**Case presentation:**

A 91-year-old African-American woman was referred to our out-patient gastroenterology clinic for evaluation of mild epigastric pain and intermittent melena. An abdominal computed tomography scan was remarkable for multiple hepatic cysts. Esophagogastroduodenoscopy revealed multiple blood clots at the ampulla of Vater. Endoscopic retrograde cholangiopancreatography showed a single 18 mm-sized filling defect in the common hepatic duct wall at the junction of the right and left hepatic duct, adjacent to one of the hepatic cysts. The ruptured hepatic cyst communicated to the bile ducts and was the cause of hemobilia with an atypical clinical presentation.

**Conclusion:**

Hemobilia is an infrequent cause of upper gastrointestinal bleeding and rarely occurs due to hepatic cyst rupture. To the best of our knowledge, this is only the second case report in the literature that describes hemobilia due to hepatic cyst rupture. However, it is the first case in the literature of hemobilia due to hepatic cyst rupture in the absence of iatrogenic or spontaneous trauma, and in the absence of a spontaneous or pathological coagulopathy.

## Introduction

Hemobilia is a rare cause of upper gastrointestinal bleeding. The widespread use of imaging modalities such as magnetic resonance cholangiopancreatography (MRCP), endoscopic ultrasound (EUS), and high-resolution computed tomography (CT) have facilitated the diagnosis of hepatic disorders and increased the indication for invasive hepatobiliary procedures. These procedures in turn increase the incidence of hemobilia. A review of hemobilia cases by Sandblom indicated that trauma was the cause of hemobilia in half the cases, with one-third of these hemobilia cases being iatrogenic, and needle biopsy of the liver representing the most common cause [1,2].

Other factors commonly associated with hemobilia include percutaneous transhepatic cholangiography (PTC), endoscopic retrograde cholangiopancreatography (ERCP), sphincterotomy, instrumental exploration of bile ducts, and biliary stent trauma. Less common causes include hepatic abscesses, cholangitis, hepatic cysts, hepatobiliary tumors, coagulopathies, and hepatic artery aneurysms that rupture [1-4]. Table 1 summarizes the various causes of hemobilia, stratified by pathophysiology. The most classic presentation of hemobilia is the triad of right upper quadrant pain, obstructive jaundice, and melena. We report a rare case of hemobilia secondary to a spontaneous hepatic cyst rupture that occurred in the absence of trauma, in the absence of coagulopathy, and in the absence of the classic triad of symptoms.

**Table 1:** Causes of hemobilia stratified by pathophysiology

Pathophysiology	Various causes of hemobilia
Iatrogenic trauma (most common cause)	Most common causes: needle biopsy of liver, percutaneous biliary drainage
	Sphincterotomy, endoscopic retrograde cholangiopancreatography (ERCP), percutaneous transhepatic cholangiogram (PTC)
	Biliary stent trauma, cholecystectomy, choledochoscopy (spyglass)

Trauma	Blunt trauma more common than penetrating trauma

Inflammation	Gallstone disease and cholangitis (most common in this category)
	Acalculous cholecystitis, polyarteritis nodosa
	Tropical hemobilia (ductal parasitism caused by *Ascaris lumbricoides*)

Hepatic polyp or neoplasm	Benign lesions such as benign adenomatous polyp, diffuse papillomatosis
	Gall bladder polyp
	Malignant neoplasms such as hepatocellular carcinoma, cholangiocarcinoma; metastasis

Spontaneous hemobilia	From pathological coagulopathy or therapeutic coagulopathy

Aneurysms	Ruptured hepatic artery aneurysm (most common in this category)
	Cystic artery pseudoaneurysm
	Post-traumatic pseudoaneurysm of an anomalous right hepatic artery with arterio-biliary fistula (rare case report)

Hepatic cystic lesions	Hepatic abscess or hepatic cyst (hemobilia complicating liver abscess and/or cyst)

Miscellaneous (rare)	Potentially fatal hemobilia due to inappropriate use of an expanding biliary stent, pancreatic pseudocyst
	Arterio-choledochal fistula, erosion of hepatic artery by cholelithiasis with cholecystoduodenal fistula

## Case presentation

**Figure 1:**
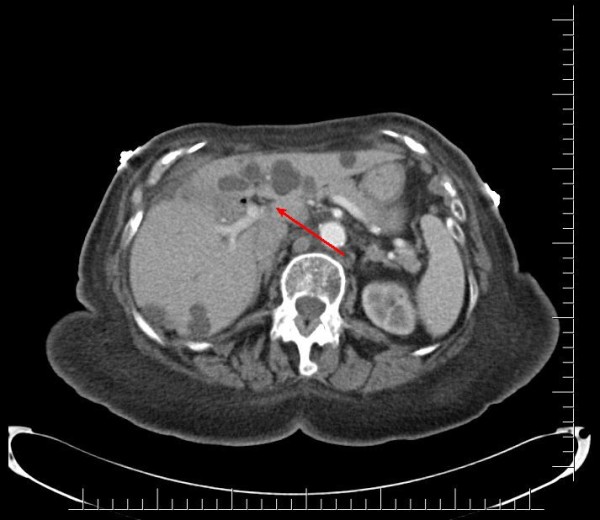
**Axial computed tomography showing a hepatic cyst infiltrating upon the common hepatic duct and causing hepatic duct dilation**.

**Figure 2:**
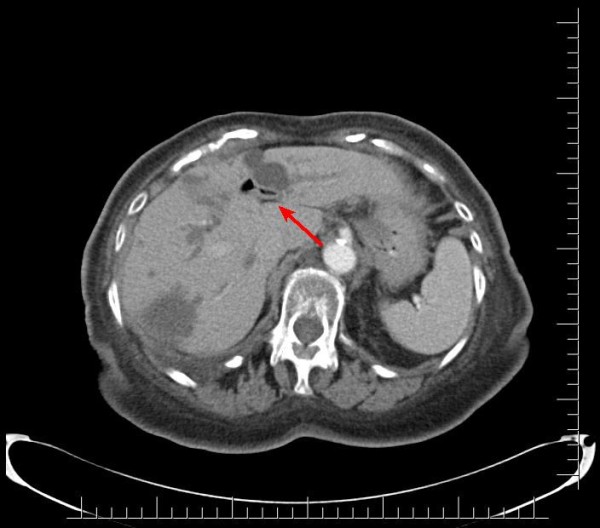
**Axial computed tomography showing a hepatic cyst infringing on the ductal system**.

**Figure 3:**
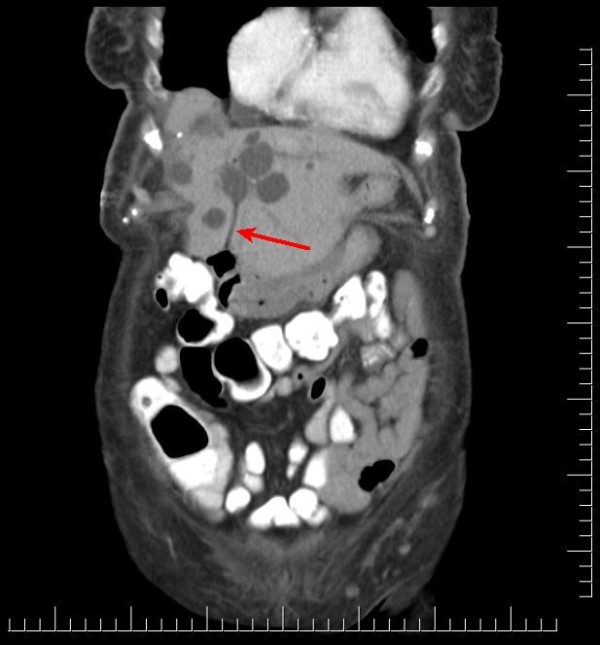
**Coronal computed tomography showing hepatic cyst communication with the common bile duct**.

**Figure 4:**
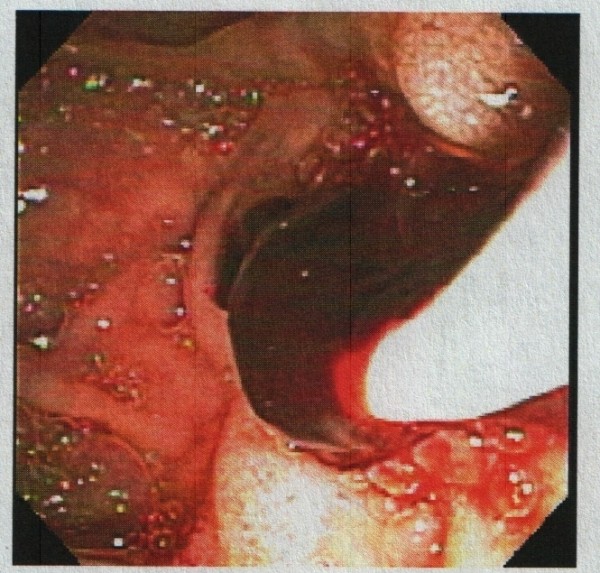
**Endoscopic retrograde cholangiopancreatography (ERCP) evaluating the 18 mm common hepatic duct filling defect, and revealing obstructing blood clots**.

**Figure 5:**
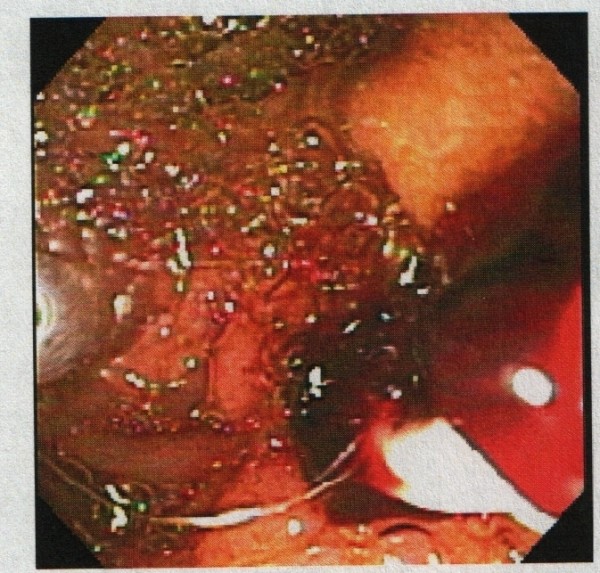
**Endoscopic retrograde cholangiopancreatographic (ERCP) removal of obstructing blood clots is initiated**.

A 91-year-old African-American woman was referred to our out-patient gastroenterology (GI) clinic for evaluation of mild epigastric pain and intermittent melena. Melena had been occurring for a week. Our patient denied any history of nausea, vomiting, early satiety, weight loss, hematemesis, heartburn, or alcohol use, and had never had any previous episodes of abdominal pain. Her vital signs showed no signs of orthostasis. An abdominal examination revealed a soft, non-tender abdomen. Rectal examination revealed hemoccult-positive dark colored stool. Laboratory test results revealed anemia (hematocrit 30%, white blood cell count 4.7 cells/mL, platelet count of 243 cells/mL) and abnormal liver function tests (LFTs; total bilirubin of 0.8 mg/dL, alkaline phosphatase of 664IU/L, aspartate aminotransferase (AST) 54 U/L, alanine aminotransferase (ALT) 188 U/L), consistent with cholestasis. Her α-fetoprotein, carbohydrate antigen (CA) 19-9, and hepatitis serologies were normal. Abdominal computed tomography (CT) studies were remarkable for multiple hepatic cysts, but malignancy could not be excluded (Figures 1,2,3). On esophagogastroduodenoscopy (EGD), the esophagus, gastric and duodenal mucosas were normal, whereas multiple blood clots were revealed at the ampulla of Vater. Hemobilia was suspected as the cause of melena and occult biliary obstruction. Our patient underwent an ERCP procedure that showed a single 18 mm large filling defect in the common hepatic duct wall at the junction of the right and left hepatic duct, adjacent to one of the hepatic cysts. The obstructing blood clots (Figures 4 and 5) were removed with a 9 mm balloon and a 7-French 10 cm stent was placed in the proximal common hepatic duct. Cytology brushing results were negative for malignancy. The presence of blood clots in the common hepatic duct (CHD) as well the adjacent localization of a hepatic cyst were strong indications that the cause of hemobilia was the hepatic cyst. Our patient's hospitalization period was uneventful and she was well one month after the intervention with no signs of hemobilia recurrence.

## Discussion

The clinical presentation of hemobilia varies tremendously. The classical triad of hemobilia symptoms, as described by Sandblom, are upper GI bleeding, right upper quadrant pain and obstructive jaundice [2]. However, most patients do not present with all of these classical findings and atypical presentations include hematemesis, epigastric pain and cholestasis without jaundice, as seen in our patient. Blood may clot in various locations depending on the amount and the rate of bleeding. Hematemesis or melena may occur if the bleeding is brisk or if the blood does not clot in the biliary tract. If blood clots within the bile duct, it may cause obstructive jaundice, cholecystitis, or pancreatitis. Our patient presented with upper gastrointestinal bleeding in the form of melena, which was relatively painless, with mild abdominal discomfort in the epigastrium. Our patient had incomplete obstruction caused by intra-biliary blood clots that caused alkaline phosphatase elevation without jaundice. This is also shown by the fact that neither the CHD or common bile duct (CBD) were dilated and there was blood at the ampulla of Vater.

Hemobilia can be classified as mild, moderate or severe, depending on the amount of blood loss and the duration of bleeding. Mild to moderate hemobilia is defined as blood loss that is less than 10% of the blood volume and thus, patients are hemodynamically stable without the need for a blood transfusion [2]. Mild to moderate hemobilia usually resolves within 48 hours without intervention. Our patient would be classified as a case of mild hemobilia, but interestingly did not have cessation of her bleeding. Severe hemobilia is diagnosed when hemorrhage constituting blood loss is greater than 10% of the total blood volume and results in hemodynamic instability that necessitates transfusion [2].

An endoscopy can confirm the diagnosis of hemobilia in only 10% of cases, which is increased slightly if the patient presents with an upper GI bleed. The role of EGD is to rule out other causes of upper GI bleeding and to attempt visualization of blood at the ampulla of Vater. ERCP can also be used for diagnosis and is more sensitive in revealing the etiology of hemobilia. Selective angiography can also be used and allows confirmation of the diagnosis of hemobilia. It should be considered early and as the test of choice in cases of severe hemobilia from trauma or known tumors for both diagnosis and therapy. Given that our patient was classified as having mild hemobilia, an angiography was not indicated. In most cases, angiography locates the vascular lesion(s) responsible for hemobilia and treatment modalities, such as therapeutic artery embolization, can be offered simultaneously. However, the diagnosis and evaluation of hemobilia [5-7] can also be facilitated by the widespread availability of imaging techniques such as abdominal US, MRCP, and high-resolution CT [8,9]. US or CT may be helpful in demonstrating intra-hepatic tumors, intra-luminal clots, biliary dilation, or hematomas responsible for the bleeding. CT may also show risk factors associated with hemobilia such as cavitating central lesions and aneurysms. MRCP can distinguish blood from stones and/or sludge and may be helpful when the diagnosis is uncertain. PTC and newer modalities such as cholangioscopy can also diagnose hemobilia.

The management of hemobilia is directed at hemostasis and relieving biliary obstruction [10,11], while replenishing the patient's blood loss by transfusions, intravenous fluids, iron, and folic acid supplements [5]. Most cases of minor hemobilia can be managed conservatively with correction of coagulopathy, adequate biliary drainage (only if necessary), and close observation, which was seen in our patient. In a recent review of 171 reported hemobilia cases from 1996 to 1999, 43% of cases were successfully managed conservatively. The first line of therapy for major hemobilia is transarterial embolization (TAE), and success rates of 80% to 100% have been reported in the most recent literature [12]. Angiography with TAE is indicated for major hemobilia requiring blood transfusions. Today, transarterial embolization is the gold standard for the management of hemobilia, and if it fails, the next step in management is surgical, which is rarely ever needed. Surgery should remain limited to extra-hepatic or gallbladder bleeding, and for TAE failure. Depending on the etiology of hemobilia, surgery involves direct exploration of the liver with possible hepatic resection, ligation and/or ballooning of the bleeding site by endoscopic intervention, aneurysm excision, cholecystectomy, and relief of bile duct obstruction. If liver damage is minimal, non-operative management may be successful, but this requires careful observation and serial arteriography.

The term hepatic cyst usually refers to a solitary non-parasitic cyst of the liver also known as a simple cyst [13]. However, cystic lesions can be classified into several categories: simple cysts, multiple cysts in the setting of polycystic liver disease (PCLD), parasitic or hydatid (echinococcal) cysts, abscesses mimicking cysts, or rarely cystic tumors [13]. Ductal cysts, choledochal cysts, and Caroli disease are categorized separately, as they entail bile duct involvement, which was not relevant in our patient. Cystic lesions of the liver can be distinguished based on their radiographic appearance and clinical presentation [13,14]. Simple cysts have a typical radiographic appearance, which consists of a thin walled homogenous low-density interior, as seen in our patient's abdominal CT results [13,14]. PCLD is associated with polycystic kidney disease (PKD) and consist of numerous hepatic cysts usually limited to the right lobe of the liver [15]. Our patient's presentation at the age of 95 along with the absence of PKD excluded PCLD despite multiple cysts at presentation [15]. Hydatid cysts have characteristic 'daughter cysts' within the main cyst, which was not consistent with our patient's radiographic appearance [13,16]. Amebic cysts and hepatic abscesses can be distinguished in the clinical presentation, which classically consists of abdominal pain, fever, and leukocytosis [13]. Cystic neoplasms of the liver can also be distinguished upon radiographic appearance, which is typically a heterogenous, multi-loculated cyst with internal septations [17]. Thus, our patient was found to have numerous simple hepatic cysts based on clinical presentation and radiographic appearance.

In fact, the most common hepatic cysts are simple hepatic cysts, which are generally considered benign [14]. Simple hepatic cysts are most often incidental radiographic findings and there is no role for treatment in these cysts [13,14]. These cysts rarely present with symptoms, but if symptoms occur, they are due to a large cyst size. Typical symptoms in this scenario would be right upper quadrant pain, early satiety, and abdominal bloating [13,14]. Medical and surgical interventions are not indicated unless severe complications develop, such as cystic rupture leading to secondary infection [13,14]. Hepatic cyst rupture, a very rare complication of a simple liver cyst, can mimic the clinical presentation of a liver abscess [13,14]. However, cyst rupture that occurs near the hepatic duct can cause hemobilia and would not cause infectious complication due to the drainage of cyst contents through the common bile duct. There is only one previously reported case in the literature where a simple hepatic cyst communicating with the hepatic duct ruptured, causing hemobilia [18]. In this report, the diagnosis was made by angiography [18], which was not indicated in our case due to our patient being classified as having mild hemobilia [11,12]. Thus, we report the second case in the literature of a ruptured hepatic cyst presenting with hemobilia, but the first case where the diagnosis was made with a multi-modality approach using EGD, ERCP, CT, pathology, and our patient's clinical course.

## Conclusion

In conclusion, hemobilia is a rare cause of upper GI bleed. Trauma and iatrogenic injuries are the most common causes of hemobilia. The absence of classical symptoms is unusual. A hepatic cyst represents an extremely unusual cause of hemobilia and is due to hepatic cyst rupture into adjacent intra-biliary ducts, which has only been reported once before in the literature. To the best of our knowledge, this is the first case of hemobilia due to a spontaneous hepatic cyst rupture in the absence of iatrogenic or spontaneous trauma and in the absence of a spontaneous or pathological coagulopathy. The diagnosis was made with a multi-modality approach using EGD, ERCP, CT, pathology, and our patient's clinical course.

## Consent

Written informed consent was obtained from the patient's next-of-kin for publication of this case report and any accompanying images. At the time of discharge, she was 91. Unfortunately, she passed away approximately 4 years later, due to unrelated causes to her case presentation. A copy of the written consent is available for review by the Editor-in-Chief of this journal.

## Competing interests

The authors declare that they have no competing interests.

## Authors' contributions

VS performed the literature review, wrote the manuscript, and provided the revised table. VS also performed the data collection, chart review, image retrieval, and incorporated all the suggested revisions including the hepatic cyst literature addendums. VS also revised all the figures, edited the images, and incorporated them in the manuscript. SD was the gastroenterologist for our patient's care and performed the endoscopy examination along with the ERCP. SD also assisted in revision of the manuscript. DA wrote a preliminary draft of the manuscript and also provided the original table. MA reviewed the manuscript. All authors read and approved the final manuscript for publication.
